# Clinical Implications of Lysyl Oxidase-Like Protein 2 Expression in Pancreatic Cancer

**DOI:** 10.1038/s41598-018-28253-9

**Published:** 2018-06-29

**Authors:** Nobutake Tanaka, Suguru Yamada, Fuminori Sonohara, Masaya Suenaga, Masamichi Hayashi, Hideki Takami, Yukiko Niwa, Norifumi Hattori, Naoki Iwata, Mitsuro Kanda, Chie Tanaka, Daisuke Kobayashi, Goro Nakayama, Masahiko Koike, Michitaka Fujiwara, Tsutomu Fujii, Yasuhiro Kodera

**Affiliations:** 1 Department of Gastroenterological Surgery (Surgery II), Nagoya University Graduate School of Medicine, Nagoya, Japan; 2 Department of Surgery and Science, Graduate School of Medicine and Pharmaceutical Sciences, University of Toyama, Toyama, Japan

## Abstract

Lysyl oxidase (LOX) family genes, particularly lysyl oxidase-like protein 2 (LOXL2), have been implicated in carcinogenesis, metastasis, and the epithelial-to-mesenchymal transition (EMT) in various cancers. This study aimed to explore the clinical implications of LOXL2 expression in pancreatic cancer (PC) in the context of EMT status. LOX family mRNA expression was measured in PC cell lines, and LOXL2 protein levels were examined in surgical specimens resected from 170 patients with PC. Higher *LOXL2* expression was observed in cell lines from mesenchymal type PC than in those from epithelial type PC. A significant correlation between LOXL2 expression and the EMT status defined based on the expression of E-cadherin and vimentin was observed in surgical specimens (*P* < 0.01). The disease-free survival and overall survival rates among patients with low LOXL2 expression were significantly better than those among patients with high LOXL2 expression (*P* < 0.001). According to the multivariate analysis, high LOXL2 expression (*P* = 0.03) was a significant independent prognostic factor for patients with PC. Additionally, LOX inhibition significantly decreased PC cell proliferation, migration, and invasion *in vitro*. In conclusion, LOXL2 expression is potentially associated with PC progression, and LOXL2 expression represents a biomarker for predicting the prognosis of patients with PC who have undergone complete resection.

## Introduction

Pancreatic cancer (PC) continues to have the worst prognosis of all the gastrointestinal malignancies. The survival rate of PC is dismal, even among patients who have undergone complete curative resection, due to the high incidence of postoperative local recurrence and distant metastasis^1,2^. The poor prognosis is attributable to the high frequency with which the cancer invades local tissues and its ability to metastasize^3^. Tumor progression has also been revealed as a product of the evolving crosstalk between cancer cells and the surrounding tumor stroma^4^. The tumor stroma consists of fibroblasts, immune cells, endothelial cells, and the extracellular matrix (ECM), and fibroblasts play a critical role in regulating solid tumor progression^5^. PC is characterized by an abundance of stromal tissue, which plays a critical role in tumor invasion, metastasis, and chemotherapy resistance^6^.

Lysyl oxidase (LOX) is an enzyme that catalyzes the cross-linking of collagen and elastin components in the ECM^7^, a process that controls both the structure and the tensile strength of the ECM and thus acts to preserve tissue integrity. The LOX family comprising the prototypic LOX and LOX-like (LOXL) proteins 1 through 4 has been implicated in carcinogenesis and metastasis. In particular, LOXL2 down-regulates E-cadherin expression and promotes the epithelial-to-mesenchymal transition (EMT) in various cancers^8–11^. As shown in our previous report, the EMT status is one of the critical prognostic factors for PC^3^, and LOXL2 interacts with SNAI1, leading to E-cadherin repression and the EMT^9^. Moreover, SNAI1 plays an important role in human PC^12^; therefore, we hypothesized that LOXL2 is a powerful regulator of the EMT and is strongly associated with PC progression.

The aim of this study was to clarify correlation between the expression of LOX family genes and the EMT status in PC cell lines and to confirm that LOXL2 expression evaluated by immunohistochemical staining is associated with clinicopathological factors and survival among patients with PC who underwent pancreatectomy. Additionally, we performed proliferation, migration and invasion assays using PC cell lines with a mesenchymal status with and without a specific inhibitor of the LOXL2 gene to evaluate the role of the LOXL2 gene in conferring malignant potential.

## Methods

### Pancreatic cancer cell lines and normal pancreatic epithelial cells

PC cell lines (HPAF-II, Capan-1, Capan-2, SW1990, TU8902, BxPC-3, COLO357, KPINL, CFPAC-1, Mpanc96, AsPC-1, Panc1, MiaPaCa-2 and TU8988) were purchased from the American Type Culture Collection (Manassas, VA). Normal human pancreatic ductal epithelial cells (HPDE6/C7) were kindly provided by Dr. Sarah Thayer (Massachusetts General Hospital, Boston, MA). Cells were maintained in RPMI-1640 medium (Sigma-Aldrich, St. Louis, MO) supplemented with fetal bovine serum (FBS) (Thermo Fisher Scientific, Waltham, MA), 100 U/mL penicillin and 100 μg/mL streptomycin (Life Technologies Corp., Grand Island, NY). All cells were cultured at 37 °C in a humidified atmosphere of 5% CO_2_. EMT status of each PC cell line was determined according to our previous report^3^.

### Clinical specimens from patients with pancreatic cancer

Surgical specimens were obtained from 170 patients with PC who underwent resection at Nagoya University Hospital in Japan between April 2004 and March 2010. In this cohort, 157 patients were diagnosed with pancreatic ductal adenocarcinoma, seven with pancreatic adenosquamous carcinoma, four with acinar cell carcinoma, and two with intraductal papillary-mucinous carcinoma. All 170 patients were deemed candidates for curative resection after undergoing meticulous preoperative workups. Extended radical resection (D2) was performed in all cases. None of the patients had received preoperative radiation or chemotherapy. The pathological diagnoses and tumor classifications were determined according to the UICC TNM classification of malignant tumors 7th edition^13^. The study was approved by the Nagoya University Hospital Ethics Committee. All patients provided written informed consent for the subsequent use of their resected tissues in this study. All clinical investigations were conducted in accordance with the principles of the Declaration of Helsinki.

### Analysis of LOX family gene expression

Levels of LOX family mRNAs were determined in PC cell lines using quantitative real-time reverse transcription PCR (qRT-PCR). Total RNA (10 μg per sample) was used to generate complementary DNA (cDNA) strands, which were amplified with specific primers for the LOX family genes (*LOX*, *LOXL1*, *LOXL2*, *LOXL3* and *LOXL4*). The reaction comprised an initial denaturation step at 95 °C for 10 minutes, followed by 40 cycles at 95 °C for 10 seconds and 60 °C for 30 seconds. All samples were tested in triplicate, and samples without templates were included on each PCR plate as negative controls. The ABI StepOnePlus Real-Time PCR System (Applied Biosystems, Foster City, CA) was used for real-time detection of the SYBR Green fluorescence emission intensity. The glyceraldehyde-3-phosphate dehydrogenase (*GAPDH*) mRNA served as the internal standard, and the expression level of each sample was calculated as the expression of the LOX family mRNA divided by the level of the *GAPDH* mRNA. The PCR primers used in the current study are shown in Supplementary Table S1.

### Publicly available dataset

Normalized RNA-sequencing data for pancreatic adenocarcinoma from The Cancer Genome Atlas (TCGA) was downloaded from the Broad GDAC Firehose (http://gdac.broadinstitute.org/, accessed on February 1st, 2018). This dataset includes 185 PC cases, including 138 cases with information on recurrence-free survival (RFS) and 163 cases with information on overall survival (OS).

### Immunohistochemistry

An anti-LOXL2 antibody (Abcam, Cambridge, UK) was used to detect the LOXL2 protein. Firstly, we deparaffinized the tissue sections, next we blocked endogenous peroxidase activity, and then heated them in Target Retrieval Solution (Agilent, Santa Clara, CA) in an autoclave for 20 minutes at 105 °C. After being cooled down, specimens were incubated with a rabbit monoclonal antibody against LOXL2 (diluted 1:250 in antibody diluent, Dako, Carpinteria, CA) for 15 minutes at room temperature. AS a horseradish peroxidase (ab202536, Dako, Carpinteria, CA) was directly labeled to the LOXL2 antibody, a secondary antibody was not required in the process. The resulting antigen-antibody complexes were visualized by exposing the samples to liquid 3,3′-diaminobenzidine (Nichirei, Tokyo, Japan) for 5 minutes. Specimens were counterstained with Mayer’s hematoxylin. EMT status of clinical PC specimen was classified according to our previous study^3^. Both EMT status and LOXL2 expression grade were determined by immunostaining. With the five different regions of microscopic view on each PC section, the cancer tissue was categorized from grade 1 through 3, as follows: grade 1, 0 to 25% staining; grade 2, 26% to 50% staining; and grade 3, more than 50% staining. The EMT status of each cancer was determined as follows: vimentin grade/E-cadherin grade < 1 was epithelial; vimentin grade/E-cadherin grade = 1 was intermediate; and vimentin/E-cadherin > 1 was mesenchymal. We then graded LOXL2 expression in each tissue section using a scale that combined the staining range and staining concentration grades (Supplementary Table S2). We defined 3 points for grade 3, 2 points for grade 2 and 2 points for grade 2, 1 point for grade 1, and calculated the sum of staining range and staining concentration scores. Combined staining range and staining concentration grades <4 considered as low LOXL2 expression, and ≥4 considered as high LOXL2 expression^14^.

### Cell proliferation, migration, and invasion assays

β-aminopropionitrile (BAPN) is an irreversible inhibitor of LOX activity^15^. As shown in the study by Yang *et al*., BAPN significantly decreases the invasive potential and the migration capacity of several cancer cells *in vitro*^16^. We performed the WST-1 cell proliferation assay (TaKaRa-Bio, Tokyo, Japan), according to the manufacturer’s instructions, to evaluate the effects of LOX inhibition by BAPN (Sigma, St. Louis, MO) on cell proliferation. Cell proliferation of the MiaPaCa-2 and Panc1 cell lines were evaluated using Cell Proliferation Reagent WST-1 (Dojindo Molecular Technologies, Inc., Kumamoto, Japan). Briefly, 1 × 10^3^ cells in 100 μl of culture medium were seeded in a 96-well plate and treated with the following concentrations of BAPN: 0, 0.2, 1 and 2 mM. After incubations for 0, 24, 48, 72 or 96 hours, 10 μl of WST-1 solution were added to each well, and the plates were incubated for 30 minutes at 37 °C. The absorbance was measured at 440 and 630 nm using a microplate reader. Each experiment was performed in three wells and was repeated independently at least three times.

Cell migration was evaluated using the wound healing assay, as previously described. Briefly, 5 × 10^5^ cells in 70 μl of culture medium were seeded in a 35-mm culture insert μ-Dish (Ibidi culture insert 35-mm dish, 81176, Germany). After 24 hours, the medium was removed, and medium containing 2 mM BAPN or a vehicle control was added to the insert. The migration of Panc-1 and MiaPaCa-2 cells into the wound area was observed after 24 and 48 hours, respectively. We assessed cell migration by measuring the area of the wound every 4 hours (×100 magnification).

The ability of PC cells to invade Matrigel was determined using BioCoat Matrigel invasion chambers (BD Biosciences, Bedford, MA), according to the manufacturer’s protocol. The assay chamber filters were hydrated and then incubated for 2 hours at 37 °C. A total of 0.75 mL of medium containing 5% FBS was subsequently added to the bottom chamber, and 2.5 × 10^4^ cells in 0.5 mL of serum-free DMEM (Sigma-Aldrich, St. Louis, MO) were added the upper chamber in each well. The chamber was incubated at 37 °C for 48 hours, during which time the cells in the upper chamber invaded the lower chamber. Cells were then fixed with 4% polysorbate and dyed with Giemsa stain. A microscope (×200 magnification) was used to count cells in eight randomly selected fields.

### Statistical analysis

The Mann-Whitney U test or Fisher’s exact test was used to determine the significance of differences between two groups, and a χ^2^ test was used to analyze the significance of associations between LOXL2 protein levels and patients’ clinicopathological parameters. The survival analysis was performed using the Kaplan-Meier method, and differences in the survival curves were evaluated using the log-rank test. All statistical analyses were performed using JMP 13 software (SAS Institute Inc., Cary, NC, USA). P values < 0.05 were considered statistically significant in all tests.

## Results

### LOX family gene expression in PC cell lines and a publicly available dataset

Firstly, levels of the *E-cadherin* and *Vimentin* (EMT markers) mRNAs were examined in 15 different PC cell lines using qRT-PCR. Each cell line was categorized as the epithelial or mesenchymal type according to *E-cadherin* and *Vimentin* expression using previously reported methods^3^. According to qRT-PCR for *LOX* family genes, only the association between *LOXL2* expression and EMT status was seen in studied PC cell lines (Supplementary Figure S1A) and the expression of other *LOX* family genes was not associated with the EMT status of PC cell lines (Supplementary Figure S1B–E).

**Figure 1:**
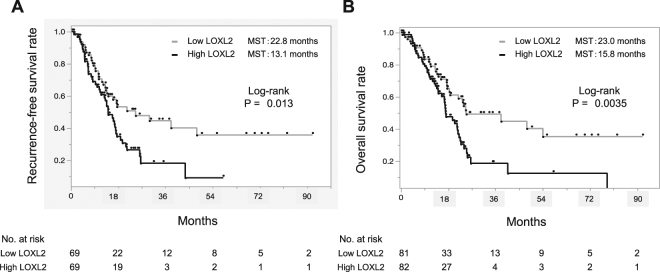
Survival analysis stratified by *LOXL2* expression levels in pancreatic cancer (PC) cases from The Cancer Genome Atlas (TGCA) datasets. (**A**) Kaplan-Meier Analysis for recurrence-free survival (RFS) stratified by LOXL2 expression. (**B**) Kaplan-Meier Analysis for overall survival (OS) stratified by LOXL2 expression. Both RFS and OS of PC cases with high *LOXL2* expression were significantly worse than the cases with low *LOXL2* according to the log-rank test.

Secondly, we confirmed the impact of *LOXL2* expression on PC prognosis using TCGA datasets. Based on the normalized RNA-sequencing data, PC cases were divided into two groups according to the *LOXL2* mRNA expression levels in PC tissues (low, <median value; high, ≥median value). As a result, both the RFS and OS of the PC cases with high *LOXL2* expression were significantly worse than PC cases with low expression (Fig. 1). In addition, we performed the *in silico* analysis with TCGA datasets to reveal the association between other LOX family genes and prognosis of PC patients (Supplementary Figure S2). This analysis revealed that LOXL2 was the only gene among LOX family genes that was able to stratify both the PC patients’ RFS and OS and we decided to employ LOXL2 for the further analyses and the experiments.

### Correlations between LOXL2 expression and clinicopathological factors in patients with resected PC

**Figure 2:**
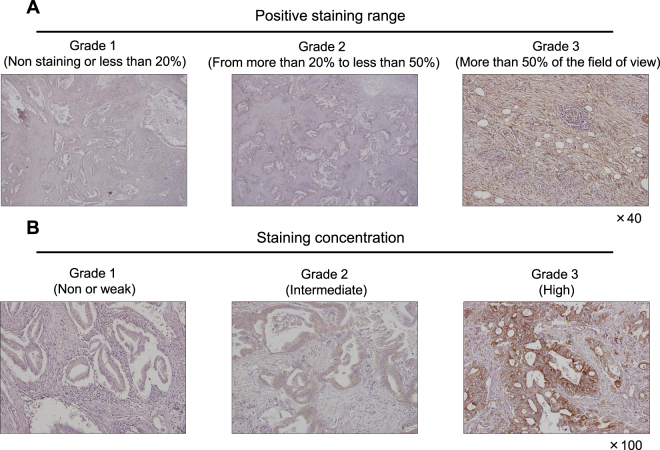
Representative images of positive LOXL2 immunohistochemical staining in surgically resected PC tissue. (**A**) Positive staining rate was defined by the area stained by LOXL2 antibody. Grade 1 is corresponded to non-stained or partially stained specimens (less than 25% of the field of view). Grade 2 indicates from 25% to 50% stained specimens and grade 3 is more than 50%. (**B**) Grading according to LOXL2 staining intensity (weak, intermediate, and strong).

We performed immunohistochemical staining for LOXL2 in surgically resected PC tissues from 170 patients to evaluate the level of the LOXL2 protein. Patients’ demographic data are shown in Table 1. Most of the patients enrolled in this study were male (102/170), had cancer in the head of the pancreas (129/170), and suffered from stage IIB cancer (88/170). The LOXL2 protein was localized in the cytoplasm of PC cells and stromal cells (Fig. 2**)**.

**Table 1:** Patients’ demographics.

Age (years), median (range)	64 (35–84)
Gender	
Male	102
Female	68
Tumor location	
Head	129
Body/Tail	36
Entire tissue	5
Operative methods	
PD	59
SSPPD	65
DP	30
TP	16
Stage	
IA	4
IB	2
IIA	31
IIB	88
IV	45
LOXL2 expression	
High	124
Low	46

Abbreviations, PD, pancreatoduodenectomy; SSPPD, subtotal stomach-preserving pancreatoduodenectomy; DP, distal pancreatectomy; TP, total pancreatectomy; LOXL2, lysyl oxidase-like protein 2.

Most patients with PC displayed high levels of the LOXL2 protein and exhibited total expression grades of 4 or higher (124/170). Table 2 shows the correlations between patients’ clinicopathological features and LOXL2 expression levels. High LOXL2 expression was significantly correlated with an older age (≥65 years) (P < 0.01), portal vein invasion (P = 0.02), peritoneal metastasis (P = 0.04), and mesenchymal marker expression (P < 0.01).

**Table 2:** Correlation between LOXL2 expression and clinicopathological factors.

	Low LOXL2 (n = 46)	High LOXL2 (n = 124)	P value
Age (≥65 vs <65 years)	14/32	70/54	<0.01
Gender (male vs female)	28/18	74/50	1
Tumor location (head vs body/tail)	10/34	95/26	0.81
CEA (≥5 ng/mL vs <5 ng/mL)	9/36	38/85	0.28
CA19-9 (≥100 U/mL vs <100 U/mL)	24/22	80/44	0.14
Tumor size (≥32 mm vs <32 mm)	33/13	100/22	0.19
Peritoneal washing cytology (positive vs negative)	8/36	19/103	0.56
Portal vein invasion (positive vs negative)	21/25	81/43	0.02
Perineural invasion (positive vs negative)	8/38	38/86	0.12
Lymph node metastasis (positive vs negative)	30/16	99/25	0.68
Stage IA/IB/IIA/IIB/IV	2/0/13/20/11	2/2/18/68/34	0.17
Recurrence site (peritoneum/liver/local/lung/lymph node/others)	5/9/13/2/9/1	31/34/46/5/19/2	0.04
EMT (mesenchymal vs epithelial)	9/37	78/46	<0.01

Abbreviations, CEA, carcinoembryonic antigen; CA19-9, carbohydrate antigen 19-9; EMT, epithelial-to-mesenchymal transition; LOXL2; lysyl oxidase-like protein 2.

### Survival analysis stratified by LOXL2 expression

**Figure 3:**
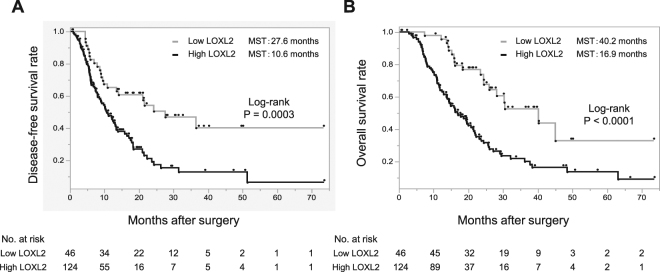
Survival analysis of in-house pancreatic cancer cases stratified by the LOXL2 expression level based on combined staining range and staining concentration grades (more than 4 was regarded as high expression). (**A**) Kaplan-Meier Analysis for Disease-free survival (DFS). (**B**) Kaplan-Meier Analysis for overall survival (OS). Both DFS and OS of cases with high LOXL2 expression were significantly worse than the cases with low LOXL2 according to the log-rank test.

Survival curves stratified by LOXL2 expression in clinically collected specimens revealed a significantly worse disease-free survival (DFS) of patients with high LOXL2 expression than in patients with low LOXL2 expression (median survival time [MST], 27.6 months; 95% Cl, 5.6–36.5 months vs. 10.6 months,; 95% Cl, 3.1–51.3 months, P = 0.0003, Fig. 3A). Similarly, the OS of patients with high LOXL2 expression was significantly worse than that of patients with low LOXL2 expression (MST = 40.2 months; 95% Cl, 15.8–73.4 months vs. 16.9 months; 95% Cl, 6.4–57.1 months, P < 0.0001, Fig. 3B).

According to the univariate analysis of DFS, the tumor location (head), tumor size (≥20 mm), mesenchymal EMT status, and high LOXL2 expression were significant prognostic factors for PC. The multivariate analysis using the Cox proportional hazard model indicated that tumor size (≥20 mm), mesenchymal EMT status and high LOXL2 expression were independent predictive factors for DFS in patients with PC (Table 3). On the other hand, the univariate analysis of OS revealed that an older age (≥65 years), gender (male), tumor location (head), tumor size (≥20 mm), portal vein invasion, perineural invasion, UICC stage, mesenchymal EMT status, and high LOXL2 expression were significant prognostic factors for PC. Based on the results of the multivariate analysis, older age (≥65 years), gender (male), tumor location (head), portal vein invasion, perineural invasion, UICC stage, mesenchymal EMT status and high LOXL2 expression were independent predictive factors for OS in patients with PC (Table 4). Thus, LOXL2 represents a robust biomarker for predicting the prognosis of patients with PC who underwent surgery.

**Table 3:** Univariate and multivariate analysis of DFS.

	Univariate		Multivariate	
	HR (95% CI)	P value	HR (95% CI)	P value
Age (≥65 vs <65 years)	1.10 (0.72–1.70)	0.65		
Gender (male vs female)	1.20 (0.78–1.86)	0.4		
Tumor location (head vs body/tail)	2.06 (1.19–3.73)	<0.01	1.07 (0.29–2.79)	0.1
CEA (≥5 ng/mL vs <5 ng/mL)	1.03 (0.65–1.64)	0.89		
CA19–9 (≥100 U/mL vs <100 U/mL)	1.13 (0.73–1.74)	0.56		
Tumor size (≥20 mm vs <20 mm)	1.85 (1.06–3.40)	0.03	1.89 (1.15–3.31)	0.03
Peritoneal washing cytology (positive vs negative)	1.87 (0.83–4.20)	0.13		
Portal vein invasion (positive vs negative)	1.03 (0.66–1.60)	0.9		
Perineural invasion (positive vs negative)	1.29 (0.81–2.05)	0.28		
Lymph node metastasis (positive vs negative)	1.73 (0.47–6.33)	0.41		
UICC Stage IA/IB/IIA/IIB/IV	1.95 (1.02–3.74)	0.05		
EMT (mesenchymal vs epithelial)	2.13 (1.35–3.42)	<0.01	2.23 (1.45–3.49)	<0.01
LOXL2 expression	1.89 (1.12–3.26)	0.02	1.88 (1.16–3.15)	<0.01

Abbreviations, CEA, carcinoembryonic antigen; CA19-9, carbohydrate antigen 19-9; EMT, epithelial-to-mesenchymal transition; LOXL2, lysyl oxidase-like protein 2; HR, hazard ratio; CI, confidence interval.

**Table 4:** Univariate and multivariate analysis of OS.

	Univariate		Multivariate	
	HR (95% CI)	P value	HR (95% CI)	P value
Age (≥65 vs <65 years)	1.78 (1.14–2.78)	0.01	1.62 (1.08–2.49)	0.02
Gender (male vs female)	2.03 (1.30–3.18)	<0.01	2.17 (1.40–3.39)	<0.01
Tumor location (head vs body/tail)	1.98 (1.13–3.72)	0.02	1.89 (1.07–3.57)	0.03
CEA (≥5 ng/mL vs <5 ng/mL)	1.21 (0.73–1.96)	0.44		
CA19–9 (≥100 U/mL vs <100 U/mL)	1.10 (0.71–1.71)	0.7		
Tumor size (≥20 mm vs <20 mm)	1.27 (0.72–2.40)	0.02	1.40 (0.81–2.56)	0.24
Peritoneal washing cytology (positive vs negative)	1.25 (0.59–2.70)	0.55		
Portal vein invasion (positive vs negative)	1.76 (1.11–2.87)	0.02	1.97 (1.25–3.16)	<0.01
Perineural invasion (positive vs negative)	1.74 (1.07–2.82)	0.03	2.70 (1.11–8.23)	0.04
Lymph node metastasis (positive vs negative)	2.52 (0.78–1.28)	0.13		
UICC Stage IA/IB/IIA/IIB/IV	1.58 (1.04–2.40)	0.04	3.40 (1.65–7.02)	<0.01
EMT (mesenchymal vs epithelial)	4.20 (2.51–7.25)	<0.01	4.65 (2.78–8.01)	<0.01
LOXL2 expression	2.07 (1.14–3.96)	0.02	1.87 (1.06–3.46)	0.03

Abbreviations, CEA, carcinoembryonic antigen; CA19-9, carbohydrate antigen 19-9; EMT, epithelial-to-mesenchymal transition; LOXL2, lysyl oxidase-like protein 2; HR, hazard ratio; CI, confidence interval.

### The effect of BAPN-mediated LOXL2 inhibition on PC cells

**Figure 4:**
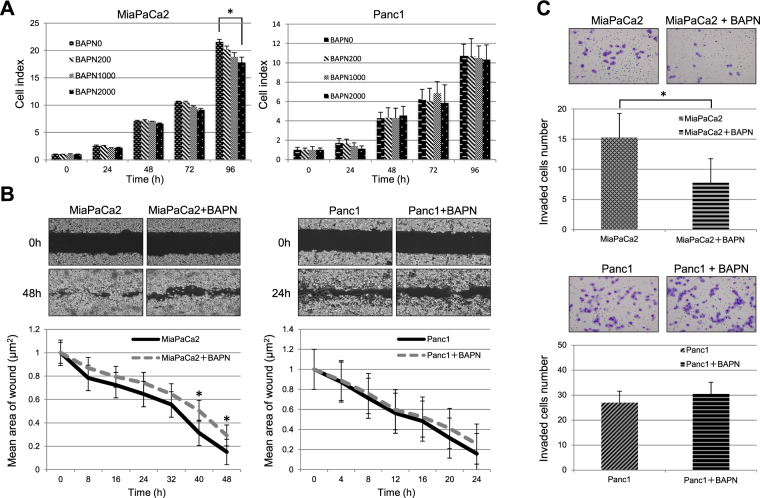
Assay of the effects of BAPN-mediated LOXL2 inhibition on MiaPaCa2 and Panc1 cells. (**A**) Cell proliferation assay using WST-1 with the different amount of BAPN. Cell index of MiaPaCa2 with BAPN after 96 hours was significantly lower with amount-dependence. * indicates statistical significance with P = 0.05 (**B**) Wound healing assay. After 40 hours, the distances of leading edge of the wound from the baseline significantly differed between BAPN free and added MiaPaCa2 but for Panc1. * indicates statistical significance with P = 0.05. (**C**) Cell invasion assay. The number of invading cells is presented as the mean numbers of invading cells from eight randomly selected fields (Å~200 magnification). BAPN significantly inhibited the cell migration of MiaPaCa cells after 48 hours of incubation though BAPN had no significant effect on Panc1 invasion. * indicates statistical significance with P = 0.05.

We evaluated the proliferation, migration and invasion of MiaPaCa2 and Panc1 cells, which exhibited high *LOXL2* expression levels, after exposing the cells to BAPN to analyze the effects of BAPN-mediated LOXL2 inhibition. The inhibition of LOXL2 expression significantly decreased MiaPaCa2 cell proliferation (P < 0.01, Fig. 4A). BAPN significantly decreased the migration of MiaPaCa2 cells in the wound healing assay (P < 0.01, Fig. 4B). Moreover, BAPN significantly decreased the number of invading MiaPaCa2 cells in the invasion assay (P < 0.01), indicating that BAPN significantly decreased the invasion ability of MiaPaCa2 cells (Fig. 4C). Based on these findings, BAPN potentially modulates LOX activity and might be used as a therapeutic tool to prevent PC invasion in further studies.

## Discussion

As reported in our previous studies, the EMT is an important intracellular biological process that plays critical roles in various cancers, including PC^3,11,17–19^. LOXL2 also reportedly interacts with SNAIL to induce the EMT, regulates FAK/Src activity and interacts with intracellular adhesion molecules, such as integrins^20^. In the present study, LOXL2 expression was significantly associated with the EMT status not only *in vitro* but also in PC tissues derived from the surgically resected specimens. Additionally, according to the survival analysis, the prognoses of patients with high LOXL2 expression were significantly worse than patients with low LOXL2 expression. Thus, LOXL2 expression represents a useful predictive biomarker for patients with PC, and LOXL2 is a putative therapeutic target for treating PC.

Although increased LOXL2 expression is reported to be associated with malignant aggressiveness in various cancer cells^9,21,22^, a very limited number of studies have examined LOXL2 expression in PC^23^. In general, clinical PC tissues tend to display enhanced LOXL2 staining in both cancer cells and the surrounding stroma. As shown in the study by Kasashima *et al*., LOXL2 secretion by fibroblasts surrounding cancer cells accelerates the invasiveness of gastric cancer cells^24^. Thus, PC invasion and metastasis are promoted not only intracellularly by the EMT but also extracellularly by desmoplastic stromal dysfunction, a phenomenon that may critically impair drug delivery^25^. Therefore, we hypothesized that LOXL2 inhibition may improve the survival of patients with PC by reducing cancer cell viability and increasing cellular sensitivity to chemotherapy.

Though several previous studies have used BAPN to inhibit LOXL2 both *in vitro* and *in vivo*^20,26–28^, we evaluated BAPN characteristics in the context of EMT status of PC cell lines. BAPN reduced the proliferation, migration and invasion of MiaPaCa-2 cells, but not Panc1 cells. MiaPaCa-2 cells do not express E-cadherin; however, they express high levels of SNAI1, a phenomenon that is correlated with highly aggressive growth and an undifferentiated state^12^. In contrast, Panc1 cells express E-cadherin and SNAI1 and are thus moderately invasive and display an epithelial growth pattern and differentiation^29^. Thus, BAPN does not appear to be effective against all PC cell lines; however, this LOXL2-targeting agent represents a potentially novel treatment option for some types of PC.

Although our study is the first to show that LOXL2 represents a putative biomarker for predicting PC prognosis and therapeutic target, some inherent limitations should be noted. Firstly, we did not compare BAPN with another known LOXL2 inhibitor, AB0023^30^, in a side-by-side experiment. Therefore, we did not clearly determine which of the two inibitors is more effective as therapeutic agent for PC. Because other members of the LOX family contribute to establishment of malignanct potential in cancer^27^, we hypothesized that BAPN, which suppresses the entire LOX family, would more effectively inhibit the unfavorable effects of LOXL2 and exclusively explored this agent. Secondly, as mentioned above, BAPN was unable to suppress Panc1 cell proliferation, migration and invasion *in vitro*. Therefore, the effects of BAPN on the expression of additional biomarkers must be analyzed to determine whether the agent is a useful therapeutic drug.

In conclusion, in the context of the EMT, LOXL2 expression is a putative biomarker for predicting the prognosis of patients with PC after complete resection and might be a therapeutic target for PC treatment. These findings might provide a substantial benefit to patients with PC, with further studies planned in the future.

## Electronic supplementary material

Supplementary materials
