# Supplementary material for: Clinical Implications of Lysyl Oxidase-Like Protein 2 Expression in Pancreatic Cancer
Source: Sci Rep. 2018 Jun 29;8:9846. doi: 10.1038/s41598-018-28253-9 (PMC6026164; doi:10.1038/s41598-018-28253-9)
Supplement: Supplementary file 1 — Supplementary materials [file 41598_2018_28253_MOESM1_ESM.pdf]

Supplementary materials

Clinical Implications of Lysyl Oxidase-Like Protein 2 Expression in Pancreatic Cancer

Authors:

Nobutake Tanaka, MD[1]; Suguru Yamada, MD, PhD[1]*; Fuminori Sonohara, MD, PhD[1]; Masaya Suenaga, MD, PhD[1]; Masamichi Hayashi, MD, PhD[1]; Hideki Takami, MD, PhD[1]; Yukiko Niwa, MD, PhD[1]; Norifumi Hattori, MD, PhD[1]; Naoki Iwata, MD, PhD[1]; Mitsuro Kanda, MD, PhD[1]; Chie Tanaka, MD, PhD[1]; Daisuke Kobayashi, MD, PhD[1]; Goro Nakayama, MD, PhD[1]; Masahiko Koike, MD, PhD[1]; Michitaka Fujiwara, MD, PhD[1]; Tsutomu Fujii, MD, PhD[2]; and Yasuhiro Kodera, MD, PhD[1]

Contents:

Supplementary table S1. Primer sets designed for RT- PCR assay in this study

Supplementary table S2. The grading of staining range and staining concentration.

Supplementary figure S1. *Lox* family gene, E-cadherin and Vimentin expression was examined in pancreatic cancer cell lines by RT-PCR. *LOXL2* expression was correlated with EMT status.

Supplementary figure S2. Survival analysis stratified by *LOX* family gene levels in pancreatic cancer (PC) cases from The Cancer Genome Atlas datasets. (A) Kaplan-Meier Analysis for recurrence-free survival (RFS) stratified by *LOX* expression. (B) Kaplan-Meier Analysis for OS stratified by *LOX* expression. (C) Kaplan-Meier Analysis for RFS stratified by *LOXL1* expression. (D) Kaplan-Meier Analysis for OS stratified by *LOXL1* expression. (E) Kaplan-Meier Analysis for RFS stratified by *LOXL3* expression. (F) Kaplan-Meier Analysis for OS stratified by *LOXL3* expression. (G) Kaplan-Meier Analysis for RFS stratified by *LOXL4* expression. (H) Kaplan-Meier Analysis for OS stratified by *LOXL4* expression.

Supplementary table S1. Primer sets designed for RT- PCR assay in this study

Primer	Sequence	Genome position	Length (bp)
LOX_F	GCGGCGGAGGAAAACTGT	964-981	19
LOX_R	AGCAGCACCCTGTGATCATAATC	1015-1037	24
LOXL1_F	GACTGCCAGTGGATCGACATAA	1875-1896	22
LOXL1_R	CTCCAAAACAATATACTTTGGGTTCA	1936-1961	26
LOXL2_F	CTCCCAGATCCACAACAATGG	2139-2159	21
LOXL2_R	AGCAGGTCATAGTGGGTGAACA	2240-2261	22
LOXL3_F	TGCAAATATGATGGACATAGAATCTG	1809-1834	26
LOXL3_R	CAAACCTCCTGTTGGCCTCTT	1870-1890	21
LOXL4_F	GGGCCCCGGGAATTATATCT	2215-2234	20
LOXL4_R	GCATATTGTTGGAGAAATCTGACTCT	2269-2294	26
GAPDH_F	GAAGGTGAAGGTCGGAGTC	19-40	19
GAPDH_R	GAAGATGGTGATGGGATTTC	69-94	20

Supplementary table S2. The grading of staining range and staining concentration.

Grade	3	2	1
Staining range	More than 50% of the field of view	From more than 20% to less than 50%	No staining or less than 20%
Staining concentration	High	Intermediate	Non or weak

Supplementary figure S1

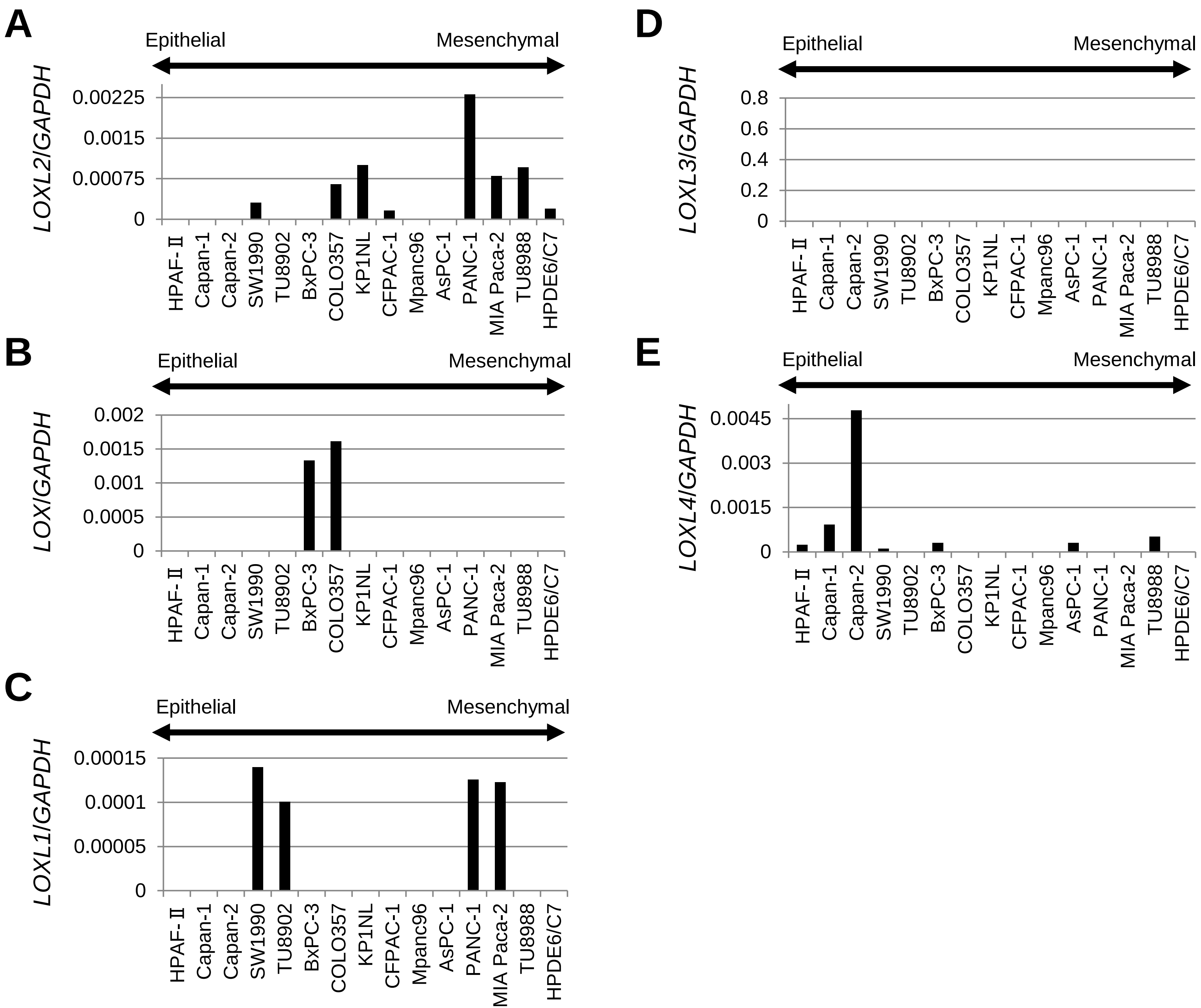

Supplementary figure S2

A

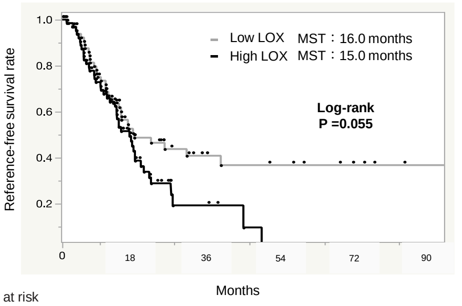

No. at risk						
Low LOX	69	23	10	7	3	2
High LOX	69	16	5	1	1	1

B

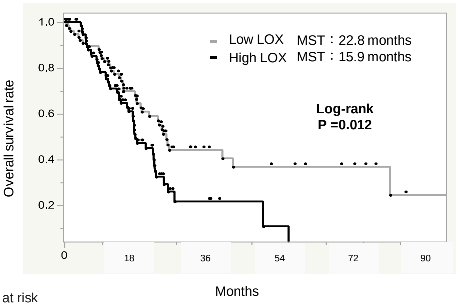

No. at risk						
Low LOX	81	33	12	8	4	1
High LOX	82	22	3	1	1	1

C

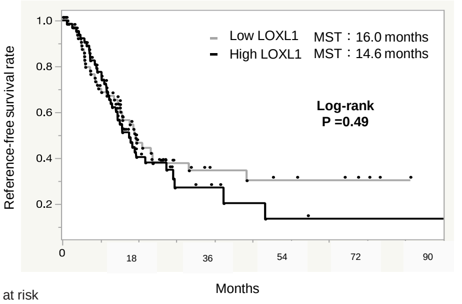

No. at risk						
Low LOXL1	69	21	9	6	2	1
High LOXL1	69	18	4	2	1	1

D

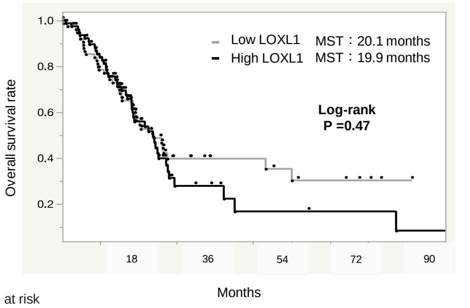

No. at risk						
Low LOXL1	81	28	10	6	2	1
High LOXL1	82	27	5	3	3	1

E

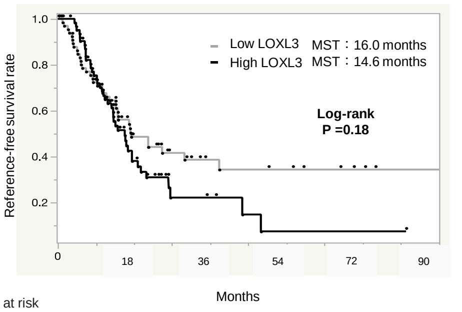

No. at risk						
Low LOXL3	69	23	9	7	2	1
High LOXL3	69	16	4	2	2	1

F

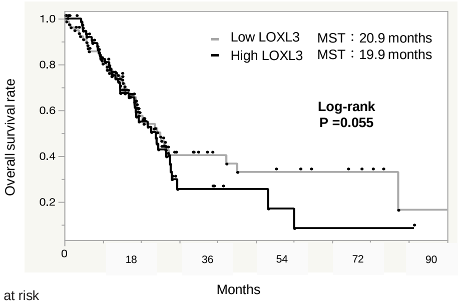

No. at risk						
Low LOXL3	81	30	11	8	3	1
High LOXL3	82	25	4	2	2	1

G

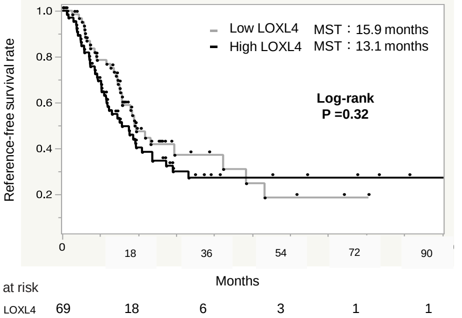

No. at risk						
Low LOXL4	69	18	6	3	1	1
High LOXL4	69	21	7	4	3	1

H

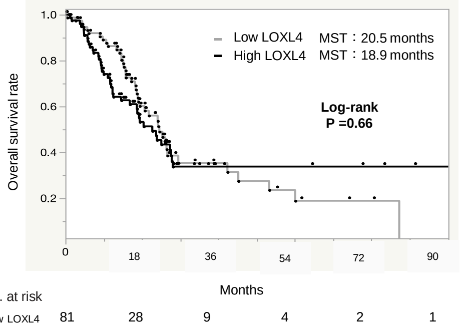

No. at risk						
Low LOXL4	81	28	9	4	2	1
High LOXL4	82	27	6	4	2	1
